# Predictors of freezing of gait in Chinese patients with Parkinson's disease

**DOI:** 10.1002/brb3.931

**Published:** 2018-02-08

**Authors:** Ruwei Ou, Qianqian Wei, Bei Cao, Wei Song, Yanbing Hou, Hui Liu, Xiaoqin Yuan, Bi Zhao, Ying Wu, Huifang Shang

**Affiliations:** ^1^ Department of Neurology West China Hospital Sichuan University Chengdu Sichuan China

**Keywords:** festination, freezing of gait, hallucination, motor fluctuation, Parkinson's disease, visuospatial impairment

## Abstract

**Objective:**

To explore the clinical predictors of freezing of gait (FOG) in Chinese patients with Parkinson's disease (PD).

**Methods:**

This study included 225 patients with PD who completed a three‐year follow‐up visit. The end‐point was the presence of FOG (freezers), which was assessed during the follow‐up visit. Group comparisons were conducted, followed by a further forward binary logistic regression analysis.

**Results:**

Eighty‐five patients with PD (38%) had developed FOG at the end of study. At baseline, freezers exhibited higher age, longer disease duration, higher scores in Unified PD Rating Scale (UPDRS) III and Hamilton Depression/Anxiety Rating Scale, lower Frontal Assessment Battery (FAB) score, higher subscores (e.g., “urgency”) and frequencies (e.g., “hallucinations”) in Non‐Motor Symptoms Scale, higher annual changes in MoCA, UPDRS III and FAB scores, and higher incidences of festination and falls than nonfreezers (*p *<* *.05). The forward binary logistic regression model indicated that a longer disease duration, a higher UPDRS III score, higher annual changes in UPDRS III score and “visuospatial/executive abilities” subscore, onset in lower limbs, and the presence of festination, falls, and hallucinations were associated with the development of FOG.

**Conclusions:**

Patients with onset in the lower limbs and the presence of festination, falls, and hallucinations may be prone to develop FOG episodes. FOG also likely occurs with the deterioration of PD severity and visuospatial function.

## 1 INTRODUCTION

Freezing of gait (FOG) is a unique and disabling clinical phenomenon in Parkinson's disease (PD), which is defined as a brief, episodic absence of or marked reduction in the forward progression of the feet despite the intention to walk (Giladi & Nieuwboer, 2008). The exact pathophysiology of FOG is poorly understood and is most likely associated with multiple brain regions involved in the locomotion (Nutt et al., 2011). FOG is a risk factor for falls for patients with PD, which results in a significant worsening of quality of life in patients with PD (Giladi, 2001). Hence, early identification of patients who are at risk of developing FOG will be valuable for patients, caregivers, and healthcare planning.

Several cross‐sectional studies have already reported the clinical correlates of FOG in PD. Our previous study found that older patients or patients with onset in the lower limbs and more severe motor disability are associated with the presence of FOG (Ou et al., 2014). An earlier American study found that FOG can occur as a result of disease progression or as a side effect of levodopa treatment (Giladi et al., 1992). Further studies found that FOG in PD is associated with executive impairment (Amboni, Cozzolino, Longo, Picillo, & Barone, 2008; Amboni et al., 2010). In addition, a recent study on autopsy‐confirmed patients with PD indicated that early cognitive impairment and hallucinations are associated with the early onset and rapid progression of FOG (Virmani, Moskowitz, Vonsattel, & Fahn, 2015).

Some prospective studies regarding the clinical predictors of FOG in PD have also been conducted. An 18‐month longitudinal seminal DATATOP study found that longer disease duration, higher nontremor score, the absence of tremor, and initial symptom as a gait disorder can predict the development of FOG in PD (Giladi et al., 2001). In another 12‐year follow‐up study, the authors found that severe disease dysfunction was not associated with developing FOG, while motor fluctuations and higher levodopa dose at baseline are independent risk factors for the development of FOG in PD (Forsaa, Larsen, Wentzel‐Larsen, & Alves, 2015). A recent three‐year follow‐up study did not find an association between levodopa treatment and future FOG episodes, but suggested that the absence of dopamine agonist receptor use is a risk factor for FOG (Zhang et al., 2016). In addition, they also found that lower educated patients and those who had a symptom of anxiety or onset of PD in lower limbs are more likely to develop FOG (Zhang et al., 2016). Based on the differences in follow‐up period, sample size, genetic background, and assessment tool, above longitudinal researches exhibit inconsistent results. To date, the clinical predictors of FOG in Chinese patients with PD are limited. Also, it is unclear whether the changes in levodopa dose and motor or nonmotor symptoms (NMSs) progression have an impact on the development of FOG in PD. More information on clinical predictors of FOG could be helpful for patients in making intervention and therapeutic strategies. Therefore, the current longitudinal study aimed to identify the differences in baseline variables, levodopa dose change, motor and nonmotor symptoms progression between patients with and without FOG, and further explore the potential clinical predictors of FOG in a large cohort of Chinese patients with PD.

## 2 METHODS

### 2.1 Patients

The study protocol was approved by the Ethics Committee of West China Hospital, Sichuan University. A total of 240 patients with PD from the Department of Neurology, West China Hospital of Sichuan University, between March 2012 and May 2013 were recruited for this longitudinal study. All participants have provided written informed consent. Inclusion criteria included (Giladi & Nieuwboer, 2008) fulfillment of the Unified Kingdom PD Society Brain Bank Clinical Diagnostic Criteria for PD (Hughes, Daniel, Kilford, & Lees, 1992), (Nutt et al., 2011) Hoehn and Yahr (H&Y) stages 1–3, and (Giladi, 2001) the absence of FOG. All patients were subjected to brain MRI scans to exclude other neurological disorders, such as stroke. Patients with atypical and secondary Parkinsonism, patients who presented with any unstable diseases, and patients who declined to be visited were excluded from the study.

**Figure 1:**
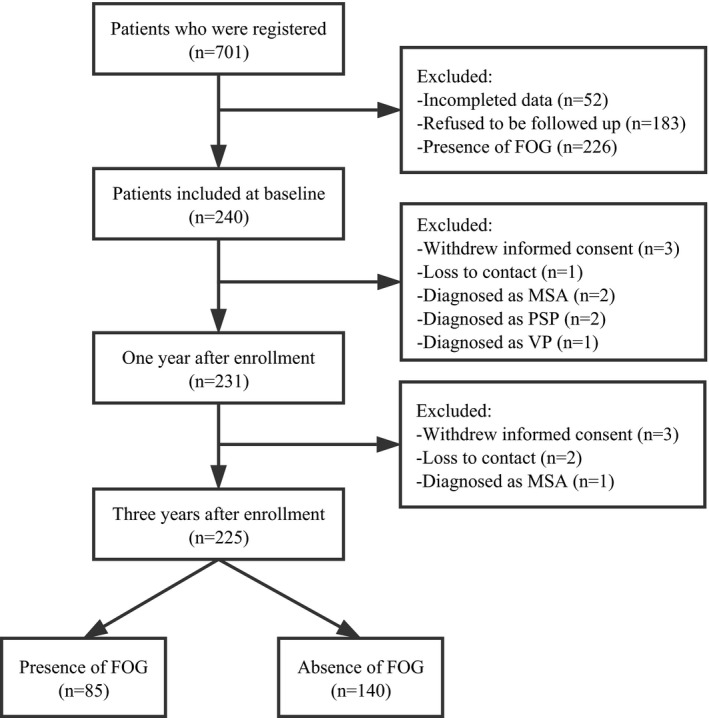
Flowchart of follow‐up for FOG

As FOG cannot occur within a short time, a follow‐up period of 3 years (range from 2.5 years to 3 years) was planned for all patients. During the follow‐up visit, assessments were not completed in nine patients who refused to return or lost contact, three patients who were diagnosed with multiple system atrophy (MSA), two patients who were diagnosed with progressive supranuclear palsy (PSP), and one patient who was diagnosed with vascular Parkinsonism (VP) (Figure 1). The remaining 225 patients were included in the data analysis.

### 2.2 Clinical assessments

At the initial visit, demographic and clinical data including age, age of onset, sex, disease duration, side and site of initial motor symptoms onset, festination, falls, medication regimen, total levodopa equivalent daily dosage (LEDD), and motor complications were collected through personal interviews conducted by neurologists who specialize in movement disorder. The total LEDD (mg/day) was calculated according to a previous systematic review (Tomlinson et al., 2010). Both the Unified PD Rating Scale (UPDRS) part III (Goetz et al., 2007) and H&Y stage (Hoehn & Yahr, 1967) were used to evaluate the motor severity. Cognitive function was evaluated using the Frontal Assessment Battery (FAB) (Rothlind & Brandt, 1993) and Montreal Cognitive Assessment (MoCA) (Nasreddine et al., 2005). Depression and anxiety were assessed using the Hamilton Depression Rating Scale (HAMD) (24 items) (Hamilton, 1967) and Hamilton Anxiety Rating Scale (HAMA), respectively (Clark & Donovan, 1994). The global NMSs were assessed using the Chinese version of Non‐Motor Symptoms Scale (NMSS) (Wang et al., 2009). All of the above‐mentioned assessments were conducted in the “OFF” medication state.

Festination was observed by trained neurologists during the visit or was reported by the patient or his/her caregiver regardless of “ON” or “OFF” medication state. If a patient with PD confirmed that his or her steps became smaller combined with a faster cadence, then he/she was identified as having festination.

Falls was defined as “unintentionally coming to rest on the ground or other lower surface without being exposed to overwhelming external force or a major internal event,” which was determined as a score ≥1 on UPDRS item 13 (falling unrelated to freezing) or a score ≥3 on UPDRS item 14 (falling related to freezing).

### 2.3 Determination of FOG

Freezing episodes were observed both by experienced neurologists and the patient himself/herself or his/her caregivers. Patients were determined to have FOG based on their response to the question “Do you feel that your feet get glued to the floor while walking, making a turn or when trying to initiate walking?” from item 1.3 of the FOG Questionnaire (Giladi et al., 2009). If patients and their caregivers could not understand the definition of FOG, a description or imitation of all of the subtypes of FOG would be performed by the neurologists to ensure the accuracy of the data. Thus, both patients with “OFF” and “ON” medication FOG were considered as freezers. Based on the assessments during the follow‐up visit, patients were divided into “freezer” group and “nonfreezer” group.

### 2.4 Statistical analyses

Continuous data were presented as mean ± standard deviation, while categorical data were presented as number (percentage). To examine whether continuous data met a normal distribution, the Shapiro–Wilk and Kolmogorov–Smirnov tests were conducted. To identify the differences in demographic and clinical features at baseline between freezers and nonfreezers groups, these variables between the two groups were compared by Student's *t*‐test, chi‐square test (or Fisher's exact test), or analyses of covariance (ANCOVA), as appropriate. For chi‐square test of 2 × 2 tables, the continuity correction was applied.

A forward binary logistic regression model was used to explore the potential clinical factors related to FOG. The presence or absence of FOG during the follow‐up visit was used as the dependent variable. The following variables that were significantly different between the two groups or had been reported previously related to FOG were used as independent variables: age, disease duration, onset in lower limbs (yes/no), use of levodopa (yes/no), LEDD, festination (presence/absence), falls (presence/absence), motor fluctuation (presence/absence), UPDRS III score, HAMD score, HAMA score, FAB score, “language”, “abstraction” and “orientation” subscores in MoCA, “urgency” subscore as well as “restless legs” (yes/no) and “hallucinations” (yes/no) in NMSS at baseline, as well as annualΔUPDRS III[ΔUPDRS = (the final UPDRS score – the initial UPDRS score) * 365/(the last visit date – the initial visit date)], annualΔvisuospatial/executive abilities score[Δvisuospatial/executive abilities score = (the initial score – the final score) * 365/(the last visit date – the initial visit date)], and annual ΔFAB score[ΔFAB score = (the initial FAB score – the final FAB score) * 365/(the last visit date – the initial visit date) ]. A collinearity test was conducted, and no collinearity existed among these variables. At the same time, a further test on the interaction between significant variables was administrated, and no interactions existed between these variables.

Statistical analyses were performed using the Statistical Package for the Social Sciences (SPSS) version 19.0. All statistical tests were two‐tailed, and *p* values <.05 were considered statistically significant.

## 3 RESULTS

A total of 225 patients with PD (129 males and 96 females) completed all prospective assessments (Figure 1), with mean age of 59.8 ± 12.3 years (range from 26.5 to 82.2 years), mean onset age of 55.4 ± 12.0 years (range from 25.0 to 79.1 years), and mean disease duration of 5.4 ± 3.0 years (range from 1.5 to 17.7 years) at enrollment. The mean UPDRS III score at baseline was 25.8 ± 12.7. During the follow‐up visit, 85 patients with PD developed FOG (38%). The baseline data of freezers and nonfreezers are presented in Table 1. At baseline, significantly higher mean age and longer mean disease duration were found in the freezers group than those in the nonfreezers group (*p *<* *.05). No significant differences in the sex distribution, mean age of onset, mean educational level, and proportion of onset in right or lower limbs were found between the freezers and nonfreezers.

**Table 1:** Baseline data of freezers and nonfreezers

	Total (*n* = 225)	Freezers (*n* = 85)	Nonfreezers (*n* = 140)	Test	*p*‐value
Education (years)	10.3 ± 4.1	9.7 ± 4.3	10.7 ± 4.0	1	.072
Sex (male, %)	129 (57%)	44 (52%)	85 (61%)	2	.239
Age (years)	59.8 ± 12.3	62.5 ± 11.1	58.1 ± 12.6	1	.009^a^
Age of onset (years)	55.4 ± 12.0	56.7 ± 11.5	54.6 ± 12.2	1	.200
Disease duration (years)	5.4 ± 3.0	6.8 ± 3.3	4.5 ± 2.4	1	<.001^a^
Onset side (right, %)	104 (46%)	34 (40%)	70 (50%)	2	.187
Onset site (lower limbs, %)	102 (45%)	46 (54%)	56 (40%)	2	.054

Test 1: Student's *t*‐test.

Test 2: Chi‐square test.

a Significant difference.

The comparisons of clinical data between freezers and nonfreezers are provided in Table 2. At baseline, the total LEDD, mean H&Y stage score, mean UPDRS III score, mean HAMD score, and mean HAMA score, as well as the proportions of levodopa use, festination and falls in the freezers group, were significantly higher than those in the nonfreezers group (*p *<* *.05), whereas the mean subscores of “language,” “abstract” and “orientation” from MoCA, and mean FAB total score in the freezers group were significantly lower than those in the nonfreezers group (*p *<* *.05). At the end of the study, the freezers group showed higher mean H&Y stage score, higher mean UPDRS III score, higher mean LEDD, higher proportion of levodopa and catechol‐O‐methyltransferase (COMT) inhibitors use, higher percentage of festination, falls and motor fluctuation, higher mean HAMD score, higher mean HAMA score, lower mean subscores for “visuospatial/executive abilities,” “language,” and “memory” as well as total score from MoCA, and lower mean FAB score compared with the nonfreezers group (*p *<* *.05). The annualΔvisuospatial/executive abilities score, annualΔMOCA score, annualΔUPDRS III score, and annualΔFAB score in the freezers group were significantly higher than those in the nonfreezers group (*p *<* *.05), whereas the remaining variables including annualΔLEDD, annualΔHAMD score, and annual ΔHAMA score between the freezers and nonfreezers were not different.

**Table 2:** Clinical features of freezers and nonfreezers

	Total(*n* = 225)		Freezers(*n *= 85)			Nonfreezers(*n *= 140)			Test	*p*‐value		
	Baseline	Follow‐up	Baseline	Follow‐up	Change (Δ^a^)	Baseline	Follow‐up	Change (Δ^a^)		*p*_1_	*p*_2_	*p*_3_
Use of levodopa	137 (61%)	201 (89%)	61 (72%)	84 (99%)	–	76 (54%)	117 (84%)	–	1	.014^a^	.001^a^	–
Use of dopamine agonists	76 (34%)	158 (70%)	31 (36%)	56 (66%)	–	45 (32%)	102 (73%)	–	1	.603	.338	–
Use of amantadine	48 (21%)	97 (43%)	18 (21%)	37 (44%)	–	30 (21%)	60 (43%)	–	1	1.000	1.000	–
Use of anticholinergic agents	24 (11%)	18 (8%)	12 (14%)	5 (6%)	–	12 (9%)	13 (9%)	–	1	.278	.510	–
Use of COMT inhibitors	8 (3.6%)	32 (14%)	5 (6%)	19 (22%)	–	3 (2%)	13 (9%)	–	1	.274	.012^a^	–
Use of MAO‐B inhibitors	2 (1%)	4 (1.8%)	1 (1%)	0	–	1 (1%)	4 (3%)	–	1	1.000	.294	–
Total LEDD (mg/day)	256.4 ± 232.5	580.9 ± 291.6	324.2 ± 230.7	694.0 ± 344.0	369.8 ± 296.4	215.2 ± 224.6	512.3 ± 230.1	297.1 ± 265.1	2	.001^a^	<.001^a^	.094
Festination	44 (20%)	67 (30%)	32 (38%)	45 (53%)	–	12 (9%)	22 (16%)	–	1	<.001^a^	<.001^a^	–
Falls	19 (8%)	31 (14%)	19 (22%)	28 (33%)	–	0	3 (2%)	–	1	<.001^a^	<.001^a^	–
H&Y stage	2.1 ± 0.6	2.5 ± 0.7	2.4 ± 0.6	2.9 ± 0.8	–	1.5 ± 0.6	2.2 ± 0.5	–	2	<.001^a^	<.001^a^	–
UPDRS III	25.8 ± 12.7	35.2 ± 14.5	32.9 ± 12.8	44.3 ± 14.8	3.7 ± 3.4	21.4 ± 10.5	29.8 ± 11.2	3.2 ± 3.0	3	<.001^a^	<.001^a^	.048^a^
Motor fluctuation	31 (14%)	63 (28%)	17 (20%)	35 (41%)	–	14 (10%)	28 (20%)	–	1	.056	.001^a^	–
Dyskinesia	10 (4%)	31 (14%)	2 (2%)	16 (19%)	–	8 (6%)	15 (11%)	–	1	.394	.131	–
MoCA	24.7 ± 4.6	24.4 ± 5.0	24.1 ± 4.8	23.2 ± 5.6	0.3 ± 1.2	25.1 ± 4.4	25.2 ± 4.4	−0.1 ± 1.2	2	.152	.008^a^	.020^a^
Visuospatial/executive abilities	3.7 ± 1.4	3.6 ± 1.5	3.6 ± 1.5	3.2 ± 1.6	0.1 ± 0.4	3.8 ± 1.3	3.8 ± 1.3	−0.0 ± 0.3	2	.338	.002^a^	.015^a^
Naming	2.6 ± 0.8	2.5 ± 0.9	2.5 ± 0.8	2.4 ± 0.9	0.1 ± 0.3	2.6 ± 0.8	2.5 ± 0.8	0.0 ± 0.3	2	.486	.219	.754
Attention	5.4 ± 1.0	5.4 ± 1.0	5.3 ± 1.0	5.3 ± 1.1	0.0 ± 0.3	5.4 ± 0.9	5.5 ± 0.9	−0.0 ± 0.3	2	.604	.163	.472
Language	2.0 ± 1.0	2.2 ± 0.9	1.8 ± 1.0	1.9 ± 1.0	−0.1 ± 0.4	2.1 ± 0.9	2.3 ± 0.8	−0.1 ± 0.3	2	.027^a^	.005^a^	.430
Abstraction	1.3 ± 0.8	1.3 ± 0.7	1.1 ± 0.8	1.2 ± 0.7	−0.0 ± 0.2	1.4 ± 0.8	1.3 ± 0.7	0.0 ± 0.3	2	.048^a^	.312	.172
Memory	3.4 ± 1.4	3.2 ± 1.7	3.3 ± 1.5	2.9 ± 1.7	0.1 ± 0.6	3.4 ± 1.4	3.4 ± 1.6	−0.0 ± 0.6	2	.931	.040^a^	.054
Orientation	5.7 ± 0.7	5.7 ± 0.8	5.6 ± 0.7	5.6 ± 0.8	−0.0 ± 0.2	5.8 ± 0.7	5.8 ± 0.8	0.0 ± 0.3	2	.019^a^	.110	.538
FAB	16.2 ± 2.1	15.5 ± 2.7	15.6 ± 2.4	14.6 ± 3.1	0.4 ± 0.7	16.5 ± 1.9	16.1 ± 2.3	0.1 ± 0.5	2	.006^a^	<.001^a^	.004^a^
HAMD	12.1 ± 9.4	11.9 ± 8.2	15.5 ± 10.0	15.4 ± 8.9	−0.1 ± 1.9	10.1 ± 8.3	9.8 ± 7.0	−0.1 ± 1.8	2	<.001^a^	<.001^a^	.771
HAMA	8.4 ± 6.4	9.0 ± 6.5	10.3 ± 6.8	11.0 ± 6.9	−0.2 ± 1.9	7.2 ± 5.8	7.7 ± 6.0	−0.2 ± 1.7	2	<.001^a^	<.001^a^	.780

COMT, catechol‐O‐methyltransferase; MAO‐B, monoamine oxidase‐B type; LEDD, levodopa equivalent daily dosage; UPDRS, Unified Parkinson's disease Rating Scale; FAB, frontal assessment battery; MoCA, Montreal Cognitive Assessment; HAMD, Hamilton Depression Rating Scale; HAMA, Hamilton Anxiety Rating Scale; ΔLEDD = the final LEDD – the initial LEDD. ΔUPDRS/ΔHAMD/ΔHAMA = (the final score – the initial score) * 365/(the last visit date – the initial visit date). ΔMoCA/ΔFAB = (the initial score – the final score) * 365/(the last visit date – the initial visit date).

Test 1: Chi‐square test or Fisher's exact test.

Test 2: Student's *t*‐test.

Test 3: ANCOVA test with adjustment for age and disease duration.

*p*_1_: comparison of baseline variables between freezers and nonfreezers.

*p*_2_: comparison of follow‐up variables between freezers and nonfreezers.

*p*_3_: comparison of change variables between freezers and nonfreezers.

a Significant difference.

The comparisons of baseline global NMS assessment between freezers and nonfreezers are listed in Table 3. The mean scores of “feelings of sadness” and “urgency” items, as well as the frequencies of “restless legs,” “lost interest in surroundings,” “lack of motivation,” “hallucinations,” “forget to do things,” “dribbling saliva,” “swallowing,” and “excessive sweating” items from NMSS in the freezers group, were significantly higher than those in the nonfreezers group (*p *<* *.05). The remaining items were not different between the freezers and nonfreezers.

**Table 3:** Baseline global NMS of freezers and nonfreezers

	NMS severity			NMS frequency		
	Freezers	Nonfreezers	*p‐*value^a^	Freezers	Nonfreezers	*p*‐value^b^
D1. Cardiovascular	1.0 ± 2.2	0.8 ± 2.0	.477	27 (32%)	34 (24%)	.285
1. Light‐headedness/dizziness	0.9 ± 1.7	0.8 ± 2.0	.492	27 (32%)	34 (24%)	.285
2. Falls because of fainting	0.1 ± 0.7	0.0 ± 0.2	.057	4 (5%)	1 (1%)	.138
D2. Sleep/Fatigue	7.4 ± 7.9	6.8 ± 7.3	.383	74 (87%)	106 (76%)	.059
3. Daytime sleepiness	1.5 ± 2.2	1.6 ± 2.3	.802	43 (51%)	60 (43%)	.322
4. Fatigue	1.8 ± 2.7	1.7 ± 2.6	.647	42 (49%)	55 (39%)	.178
5. Difficulty falling asleep	2.6 ± 3.6	2.4 ± 3.3	.468	52 (61%)	69 (49%)	.110
6. Restless legs	1.6 ± 3.0	1.1 ± 2.4	.197	34 (40%)	34 (24%)	.019^c^
D3. Mood/Apathy	11.9 ± 13.5	7.4 ± 10.0	.021^c^	62 (73%)	87 (62%)	.130
7. Lost interest in surroundings	1.9 ± 2.5	1.3 ± 2.1	.174	47 (55%)	50 (36%)	.006^c^
8. Lack of motivation	2.0 ± 2.7	1.4 ± 2.3	.158	42 (49%)	47 (34%)	.027^c^
9. Feelings of nervousness	1.9 ± 3.1	1.1 ± 2.0	.322	30 (35%)	46 (33%)	.819
10. Feelings of sadness	3.1 ± 3.1	1.7 ± 2.3	.001^c^	50 (59%)	70 (50%)	.251
11. Flat mood	1.4 ± 2.4	0.8 ± 1.8	.093	23 (27%)	34 (24%)	.760
12. Difficulty experiencing pleasure	1.6 ± 2.6	1.1 ± 2.0	.580	30 (35%)	49 (35%)	1.000
D4. Perceptual problems/Hallucinations	0.6 ± 3.7	0.4 ± 2.3	.599	19 (22%)	12 (9%)	.007^c^
13. Hallucinations	0.2 ± 1.3	0.2 ± 1.2	.453	16 (19%)	4 (3%)	<.001^c^
14. Delusions	0.1 ± 1.3	0.2 ± 1.1	.288	5 (6%)	1 (1%)	.060
15. Double vision	0.3 ± 1.2	0.1 ± 0.4	.053	10 (125)	9 (6%)	.251
D5. Attention/memory	3.6 ± 4.7	3.3 ± 4.0	.926	63 (74%)	91 (65%)	.201
16. Concentration	0.7 ± 1.4	0.7 ± 1.5	.379	28 (33%)	32 (23%)	.133
17. Forget things or events	1.9 ± 2.4	2.1 ± 2.5	.436	55 (65%)	85 (61%)	.648
18. Forget to do things	1.0 ± 1.9	0.5 ± 1.3	.072	35 (41%)	33 (24%)	.008^c^
D6. Gastrointestinal	2.4 ± 4.5	2.5 ± 3.8	.373	51 (60%)	53 (38%)	.002^c^
19. Dribbling saliva	0.5 ± 1.5	0.4 ± 1.2	.742	20 (24%)	13 (9%)	.006^c^
20. Swallowing	0.4 ± 1.2	0.2 ± 0.7	.410	17 (20%)	13 (9%)	.037^c^
21. Constipation	1.5 ± 3.3	1.9 ± 3.1	.118	36 (42%)	41 (29%)	.063
D7. Urinary	4.0 ± 6.5	4.6 ± 6.8	.648	50 (59%)	70 (50%)	.251
22. Urgency	1.2 ± 2.5	0.8 ± 2.3	.023^c^	26 (31%)	23 (16%)	.020^c^
23. Frequency	1.1 ± 2.5	1.3 ± 2.7	.881	23 (27%)	32 (23%)	.582
24. Nocturia	1.7 ± 2.7	2.5 ± 3.2	.132	40 (47%)	61 (44%)	.710
D8. Sexual dysfunction	4.0 ± 7.4	3.7 ± 7.7	.472	33 (39%)	54 (39%)	1.000
25. Interest in sex	1.9 ± 3.7	2.0 ± 3.9	.974	29 (34%)	50 (36%)	.921
26. Problems having sex	2.2 ± 4.0	1.8 ± 3.9	.174	29 (34%)	40 (29%)	.486
D9. Miscellaneous	4.4 ± 5.9	3.8 ± 4.4	.881	62 (73%)	82 (59%)	.042^c^
27. Pain	1.9 ± 3.1	1.4 ± 2.6	.055	37 (44%)	49 (35%)	.256
28. Taste or smell	1.2 ± 2.2	1.2 ± 2.2	.923	30 (35%)	40 (29%)	.364
29. Weight change	0.1 ± 0.7	0.2 ± 0.5	.387	7 (8%)	11 (8%)	1.000
30. Excessive sweating	1.3 ± 2.7	1.1 ± 2.3	.567	31 (36%)	31 (22%)	.029^c^
Total NMSS	39.0 ± 31.9	33.3 ± 29.7	.116	84 (99%)	131 (94%)	.094

NMS, nonmotor symptoms; NMSS, Non‐Motor Symptoms Scale.

a *p*‐value was calculated from Student's *t*‐test.

b *p*‐value was calculated from chi‐square test or Fisher's exact test.

c Significant difference.

The potential clinical factors related to FOG are presented in Table 4. The forward stepwise binary logistic regression model indicated that a longer disease duration (OR = 1.201, *p *=* *.007), a higher UPDRS III score (OR = 1.076, *p *<* *.001), a larger annualΔUPDRS III score (OR = 1.157, *p* = .011), a higher annualΔvisuospatial/executive abilities score (OR = 3.841, *p *=* *.012), lower limbs as onset site (OR = 2.632, *p* = .013), and the presence of festination (OR = 2.940, *p *=* *.024), falls (OR = infinite, *p *<* *.001), and hallucinations (OR = 5.407, *p *=* *.009), were associated with the presence of future FOG.

**Table 4:** Independent predictors of FOG

	OR	95% CI	*p*‐value
Disease duration	1.201	1.050–1.373	.007
Lower limbs as onset site	2.632	1.228–5.640	.013
Festination	2.940	1.152–7.501	.024
Falls	Infinite	–	<.001
UPDRS III	1.076	1.037–1.116	<.001
ΔUPDRS III	1.157	1.034–1.294	.011
Hallucinations	5.407	1.534–19.059	.009
Δ visuospatial/executive abilities	3.841	1.345–10.965	.012

FOG, freezing of gait; UPDRS, Unified Parkinson's disease Rating Scale; FAB, frontal assessment battery.

ΔUPDRS = (the final UPDRS score – the initial UPDRS score) * 365/(the last visit date – the initial visit date).

Δ visuospatial/executive abilities = (the initial score – the final score) * 365/(the last visit date – the initial visit date).

*p*‐value was calculated from binary logistic regression analysis.

## 4 DISCUSSION

This prospective study investigated the clinical predictors associated with the development of FOG based on a large cohort of Chinese patients with PD. We found that FOG develops as PD progresses and likely occurs with the deterioration of visuospatial function. We also found that patients with onset in lower limbs or the presence of festination, falls, and hallucinations may be prone to develop FOG in PD.

Our previous cross‐sectional study (Ou et al., 2014) has found that patients with onset in lower limbs were associated with the presence of FOG. In the current study, we verified that patients with onset in lower limbs were likely to develop FOG episodes, which is in accordance with the finding of another prospective study from China (Zhang et al., 2016). The pathophysiological mechanism of FOG episodes is poorly understood. Difficulty in switching between motor programs is a proposed cause of FOG in PD. A recent study (Lohnes & Earhart, 2012) implies that individuals with PD experienced movement‐switching deficits in the lower limbs, which may result in the episodes of FOG.

In the current study, we found that patients with longer disease duration are more likely to develop FOG episodes. This is consistent with the findings of a previous prospective study (Giladi et al., 2001) and several cross‐sectional studies (Garcia‐Ruiz, del Val, Fernandez, & Herranz, 2012; Giladi et al., 1992; Macht et al., 2007), but is not consistent with the results from two other prospective studies (Forsaa et al., 2015; Zhang et al., 2016). Our study also found that higher disease severity is a risk factor for FOG. Most prospective studies (Giladi et al., 2001; Zhang et al., 2016) and cross‐sectional studies (Giladi et al., 1992; Macht et al., 2007; Ou et al., 2014) agreed that patients with more severe motor disability have increased risk in developing FOG episodes, while one prospective study disagreed (Forsaa et al., 2015). In addition, we found that FOG develops as PD progresses, which is in keeping with the acknowledgment that FOG is one kind of motor phenotype in PD. However, we cannot conclude a causal relationship between FOG episodes and the deterioration of PD, as it is possible that FOG can appear firstly and in return result in the UPDRS III change. Furthermore, in the current study, our results showed that the lack of associations between the development of freezing episodes and sex as well as age. These confirm the results of three prospective studies (Forsaa et al., 2015; Giladi et al., 2001; Zhang et al., 2016), although a 10‐year clinic‐based follow‐up study does not support our findings (Garcia‐Ruiz et al., 2012). Differences in follow‐up period, sample size, genetic background, and assessment tools may contribute to such discrepancies.

The relationship between FOG and dopaminergic medication is complicated. Most FOG episodes occur in “OFF” medication state and disappeared after dopaminergic replacement therapy (Giladi et al., 1992, 2001; Macht et al., 2007). However, the FOG phenomenon is not always treatment responsive. It is reported that some FOG episodes occur in the “ON” medication state, which may be caused by imperfect, uneven dopamine supplementation (Ambani & van Woert, 1973). Moreover, a recent study found that FOG could deteriorate with dosage of levodopa increasing (Espay et al., 2012). Above evidence indicates that the pathophysiology between patients with “ON” and “OFF” FOG episodes may be different. In the current study, our findings support that the development of FOG has no relationship with levodopa treatment. First, the LEDD dose changes were not significantly different between patients with and without FOG. Second, both levodopa use and LEDD were not associated with future freezing episodes. Third, the occurrence of FOG was not associated with motor fluctuation. In addition, there is a subtype of “unresponsive” FOG, which is not impacted by dopaminergic agents and occurs both in the “ON” and “OFF” states. Our limitation was that we did not differentiate the “ON” and “OFF” FOG episodes. Further stratified study will help to verify the relationship between dopaminergic treatment and freezing episodes.

Even though the association between FOG and festination/falls has been identified by previous observational studies (Morris, Iansek, & Galna, 2008; Ou et al., 2014), our study, for the first time, demonstrates the causal relationship between freezing episodes and festination/falls. Our result indicates that these gait disturbances likely share a common pathological pathway. Festination in PD has been hypothesized to be associated with deficits in motor cue production in the globus pallidus (Iansek, Huxham, & McGinley, 2006), which can delay the time of phasic motor cues from the internal globus pallidus to the supplementary motor area and premotor cortex. When the phasic cues are extremely slow or absent, freezing occurs because the motor cortical regions are not provided with phasic cues that are necessary to enable them to generate force for the next step in the sequence.

In addition, the association between more rapid deterioration of executive function and visuospatial deficits and the development of FOG are similar to the findings of several observational studies (Amboni et al., 2008, 2010; Nantel, McDonald, Tan, & Bronte‐Stewart, 2012), which found that FOG in PD is associated with cognitive decline particularly executive dysfunction and visuospatial deficits. There is a wealth of reports showing that increased cognitive load can induce freezing behavior (Yogev‐Seligmann, Hausdorff, & Giladi, 2008), implying that there may exist a commonality between the neural networks underlying such cognitive processes and the phenomenon of freezing. A resting‐state functional magnetic resonance imaging study found that the connectivity disruption of “executive‐attention” and visual neural networks is associated with FOG in PD (Tessitore et al., 2012). Another voxel‐based morphometry study found that PD patients with FOG showed frontal and parietal atrophy, also suggesting that executive dysfunction and perception deficits may be involved in the development of FOG in PD (Kostic et al., 2012).

Finally, we found that the presence of hallucinations is an independent risk factor for PD patients with FOG, which verifies the findings of two observational studies (Factor et al., 2014; Virmani et al., 2015). It indicates that earlier cortical involvement could lead to gait impairment because numerous nonmotor features of PD are thought to be related to the presence of Lewy bodies in the cortex (Virmani et al., 2015). Whether a shared pathophysiology exists between FOG episodes and hallucinations remains to be determined. However, our findings do imply that patients with FOG need cautious monitoring, particularly if hallucinations occur.

Some limitations should be discussed. First, the establishment of FOG in the current study was based on the self‐reported questionnaire, so freezers exhibiting very mild episodes may be misdiagnosed as nonfreezers based on clinical observation, which may result in selective bias. Second, the current period of follow‐up is relatively short so that some patients may not have developed freezing episodes by the end of the study. Third, we cannot identify the clinical predictors for different phenotypes of FOG, as we did not identify the “OFF” and “ON” medication state FOG at present. Forth, “visuospatial/executive” item from MoCA as a tool to test visuospatial cognition is not comprehensive neuropsychological battery of visuospatial cognition.

## 5 CONCLUSIONS

Freezing of gait likely occurs with the deterioration of PD severity and visuospatial function. Patients with onset in the lower limbs and presence of festination, falls, and hallucinations may be prone to develop FOG.

## CONFLICT OF INTEREST

The authors declare that they have no conflict of interest.
